# Research Progress in Vitamin A and Autism Spectrum Disorder

**DOI:** 10.1155/2021/5417497

**Published:** 2021-12-07

**Authors:** Zhonghui Liu, Jingyu Wang, Qu Xu, Qin Hong, Jiansheng Zhu, Xia Chi

**Affiliations:** ^1^Women's Hospital of Nanjing Medical University, Nanjing Maternity and Child Health Care Hospital, 123 Tianfei Alley, Mochou Road, Nanjing 210004, China; ^2^Institute of Pediatrics, Nanjing Medical University, 72 Guangzhou Road, Nanjing 210008, China

## Abstract

Autism spectrum disorder (ASD) is a highly heterogeneous neurodevelopmental disorder. Over the past few decades, many studies have investigated the effects of VA supplementation in ASD patients and the relationship between vitamin A (VA) levels and ASD. VA is an essential micronutrient that plays an important role in various systems and biological processes in the form of retinoic acid (RA). Recent studies have shown that serum VA concentration is negatively correlated with the severity of ASD. The lack of VA during pregnancy or early fetal development can affect brain development and lead to long-term or even permanent impairment in the learning process, memory formation, and cognitive function. In addition, VA deficiency has been reported to have a major impact on the gastrointestinal function of children with ASD, while VA supplementation has been shown to improve the symptoms of ASD to a certain extent. This paper provides a comprehensive review of the relationship between VA and ASD.

## 1. Introduction

Autism spectrum disorder (ASD) is a pervasive neurodevelopmental disorder characterized by social interaction deficits and stereotyped and repetitive patterns of behavior, interests, and activities, as well as communication impairment [1]. ASD usually begins at or before the age of 3 [2, 3], and in the United States, approximately 1 in 59 children is diagnosed with ASD by the age of 8 [4]. Over the last few decades, the prevalence of ASD has risen significantly worldwide and has brought a tremendous burden on individuals, families, and society.

Numerous studies on the etiology of ASD have been reported to date. One of the widely accepted views is that genetic and environmental factors contribute to the development of ASD [5]. Indeed, genetic factors have been considered major etiological factors in the development of ASD all along. However, despite the advances in sequencing technologies and bioinformatic analysis techniques that have led to the identification of several candidate genes involved in ASD, all identified genetic risk factors to date account for only 10-20% of the total number of ASD patients [6]. Furthermore, the finding that individuals with several genetic risk factors for ASD do not always develop the disorder indicates that the gene mutation(s) can increase the risk of ASD, but additional risk elements are necessary. From the perspective of environmental factors, some studies have shown that the pathogenesis of autism is closely related to micronutrient deficiency, including vitamin A (VA), vitamin B, and vitamin D (VD) [7]. In recent years, the role of micronutrients, especially VA, in the pathophysiology and treatment of ASD has increasingly attracted the attention of researchers.

## 2. Vitamin A, Metabolism, and Physiology

VA is one of the essential nutrients for humans and represents a group of unsaturated nutritional organic compounds that includes retinol, retinal, RA, and multi-VA carotenoids [8, 9]. VA exists in various forms rather than a single compound. In animal foods, VA is mainly present in the form of esters, among which retinyl palmitate is the main form, which is converted into retinol in the small intestine. In plants, VA exists in the form of carotene, which is the most biologically active and ubiquitous provitamin A carotenoid [10, 11]. After being digested, VA is processed into chylomicrons by intestinal cells, metabolized by lipoprotein lipase in the duodenum, and eventually absorbed by small intestinal mucosal cells [10, 12, 13]. Subsequently, VA is transported to the liver through the blood. Approximately 70% of the VA in the liver is stored in hepatic stellate cells in the form of retinyl palmitate, and the rest is distributed to various tissues of the body. Stored retinol is released into the bloodstream from hepatic cells and binds to plasma retinol-binding protein 4 (RBP4) produced by hepatocytes. This compound is the major circulating form of VA on an empty stomach [12] and is carried by transthyretin (TTR), which forms a complex with RBP4 and reduces renal excretion of VA [14]. The transport of retinol by the retinol complex (consisting of RBP4, TTR, and retinol) to extrahepatic tissues seems to be related to the cellular receptor of RBP4 [15, 16].

VA is an essential micronutrient that functions through the active metabolite retinoic acid (RA). VA plays a significant role in the nervous, digestive, immune, and serotonin systems. In the nervous system, VA can affect brain development, the learning process, memory formation, and cognitive function by regulating the growth, proliferation, and differentiation of neurons. In the digestive system, VA can regulate intestinal bacteria and maintain the integrity of the intestinal mucosal epithelium by regulating gastrointestinal (GI) function. In the immune system, VA can regulate the expression of CD38 to modulate the secretion of oxytocin, which affects social memory, social communication, and coordination ability. Additionally, VA also plays a critical role in the serotonin system by modulating serotonin or 5-hydroxytryptamine (5-HT). Therefore, the role of VA is extremely important *in vivo*.

## 3. Vitamin A Levels in ASD Patients

Multiple studies have shown that individuals with ASD have lower levels of VA compared to typically developing (TD) children or family members [17–21]. An assessment of nutritional intake for 3 consecutive days in 367 ASD children aged 2 to 11 years in the United States (National Health and Nutrition Examination Survey data and matched subsets based on age, sex, family income, and race/ethnicity (*N* = 252 analyzed food records)), using a developing country information system, found that ASD children aged 4 to 8 years had significantly lower VA intake than controls [22]. The study found food sources of VA below the recommended intake in a large, geographically diverse cohort of ASD children, confirming previous reports of inadequate VA intake. A study in Heilongjiang Province (China) compared 53 ASD children with 53 TD children [17]. As in the abovementioned research conducted in the United States, the evaluation of the dietary nutritional intake, nutritional data, anthropometric data, biochemical assessment, malnutrition physical examination, and parental questionnaire was included in the study to further improve the nutritional assessment method. Another study included 154 ASD children (age = 5.21 ± 1.83 years) and 73 TD children (age = 4.83 ± 0.84 years) in Chongqing, China [18] and assessed the severity of ASD using the Childhood Autism Rating Scale (CARS) in addition to measuring the concentrations of serum ferritin, folic acid, vitamin B_12_, 25 (OH) VD, and VA. In all participants, they underwent anthropometric examination, dietary assessment, and questionnaire assessment regarding their eating behavior and GI symptoms. The study found that ASD children had the highest rate of VA deficiency (VAD), and the VA concentration was negatively correlated with the CARS score, a diagnostic scale completed by caregivers of ASD children to assess the severity of ASD [23, 24]. The researchers also used indirect reference data from the Children's Hospital of Chongqing Medical University database. On the basis of the anthropometric examination, dietary assessment, eating behavior, and questionnaire assessment of GI symptoms, we use the CARS and measure the relevant indicators in the serum of ASD children to obtain more compelling results. A study in Hainan Province (China) included 274 ASD children and asked them to complete the Autism Behavior Checklist (ABC) [25], Social Responsiveness Scale (SRS) [26], and Gesell Developmental Schedules (GDS) [27] to evaluate the symptoms of the patients [19]. Their study compared the vitamin and mineral levels of all ASD children and 97 TD children of similar age. Consistent with previous findings, the VAD rate was the highest in ASD children. In addition, the serum retinol levels were measured by high-performance liquid chromatography (HPLC) in both groups. The results suggested that serum VA deficiency in ASD children may be due to picky eating patterns and resistance to new foods compared to TD children. Additionally, they also found that the more severe the VAD was, the more severe the clinical symptoms were. VA was correlated with the CARS score, ABC score, and ABC subscale score, including sensory, social withdrawal, and stereotyped behavior scores. These experimental findings are useful for the development of a timely and targeted clinical intervention to prevent the occurrence of ASD. In a recent study of 323 ASD children and 180 healthy controls, autistic-like symptoms were evaluated using the CARS, SRS, and ABC and serum retinol levels were measured by HPLC [20]. The VA level, which was found to be lower in the ASD group, was related to the severity of ASD. Furthermore, this study showed that ASD children with total GI symptoms have significantly lower levels of serum VA than those without GI symptoms, and ASD children with constipation have significantly lower VA levels than those without. These results showed that VA levels were closely associated with the occurrence of GI symptoms, especially constipation in ASD children.

Previous studies were limited to the nutritional status of ASD children in a single region, but a recent study recruited 738 children with ASD and 302 TD children from Chongqing and Hainan (China) to compare their nutritional status and symptoms and analyze their response to nutritional levels and ASD symptoms [21]. They used the ABC, SRS, and CARS to evaluate the symptoms of ASD children and the GDS to assess neurodevelopment in ASD children. In addition, the nutritional status was assessed by anthropometric measurements, biochemical tests for micronutrients, questionnaires provided to caregivers, and a food frequency questionnaire (FFQ). The experimental results confirmed the occurrence of VAD in ASD children, and serum VA and VD concentrations were negatively correlated with symptom severity. These results may be explained by the tendency of ASD children in these two regions to refuse most foods rich in VA and VD. Moreover, the nutritional status of ASD children was more severe in Chongqing compared to Hainan Province; that is, there were regional differences in the nutritional status and symptoms of ASD children. This study not only comprehensively evaluated the nutritional status and symptoms of ASD children but also compared and analyzed the experimental results of children from two different regions, which not only improved the reliability of the experimental results but also provided a valuable reference for future large-scale, multicenter collaborative research.

The above evidence suggests that the levels of VA are lower in ASD children compared with TD children and that the severity of ASD-related symptoms is negatively correlated with the levels of VA.

## 4. The Mechanisms of VAD in ASD

Numerous studies have suggested that VAD may be associated with the etiology of ASD. The possible mechanisms are as follows: (1) VA may affect nervous system function by acting on synapses, the hippocampus, the striatum, and other brain structures; (2) VA may affect digestive system function by regulating intestinal bacteria and intestinal lymphoid tissue; (3) VA may reduce oxytocin release by regulating the expression of CD38, thereby affecting the symptoms of ASD; and (4) VA can improve ASD-like symptoms by regulating 5-HT levels.

### 4.1. VAD in the Nervous System of ASD

**Figure 1:**
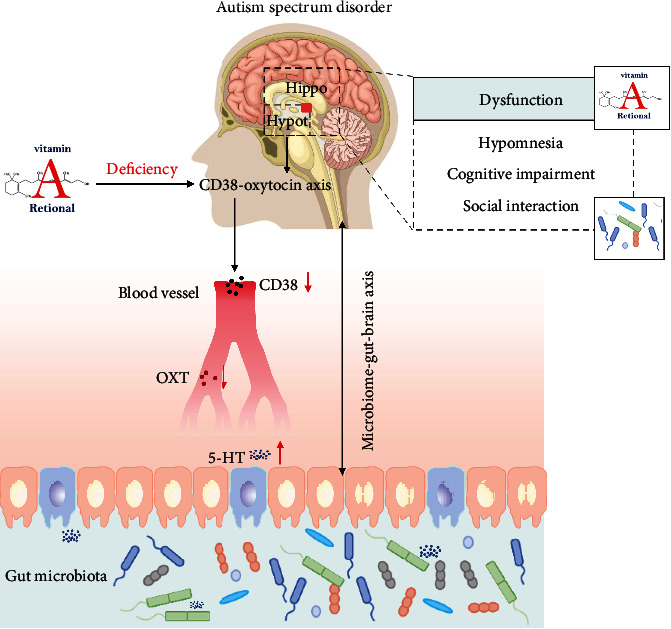
The multiple effects of VA in ASD. In the nervous system, VAD can lead to amnesia, cognitive impairment, and social impairment. In the GI system, VAD affects GI bacteria, causing associated symptoms. In the immune system, VAD can lead to a decrease in serum 5-HT and OXT through the CD38-OXT pathway. In addition, the nervous system and the GI system can interact with each other by modulating the microbiota-gut-brain axis.

Many studies have shown that VAD affects nervous system development, leading to nervous system-related diseases, such as Alzheimer's disease [28], multiple sclerosis [29], Parkinson's disease [30], and fragile X syndrome [31]. As an essential fat-soluble vitamin, VA mainly affects neural patterning [32, 33], neuronal differentiation [34], neurite growth, and axonal elongation through its derivative RA, which influences the development of the central nervous system [35]. The neural elements that may underlie the pathophysiology of ASD are well known and may include regions, such as the cerebellum, hippocampus, and cortex [8, 36], where RA receptors have been found to be highly expressed [37, 38]. This suggests that RA may be linked to ASD by impacting these regions. The hippocampus is an important organ for memory processing, storage, and spatial information processing in the brain, and impairment of its functions may be irreversible if a deficiency of VA supplementation is initiated during a critical period of hippocampal development. In the hippocampus, VAD can affect synaptic plasticity in hippocampal neurons, thereby hindering the learning process and memory formation, and can impair cognitive function by inhibiting the activity of calcium ion-dependent proteins in hippocampal neurons [39], leading to an ASD phenotype [40]. Experimental data showed that the expression of dopamine-related genes (*Drd1*, *Drd2*, *Drd5*, and *Dat*) and hypermethylation of the promoter region of the *Drd2* gene were higher in the hippocampus of male rat offspring fed a 10-fold higher VA diet. In addition, the same dose (10-fold of VA) fed to rat offspring in the postweaning diet also increased the expression of *Drd2* and *Drd5* genes in the hippocampus [41, 42]. Also, the results of another study suggest that VAD during pregnancy may be a risk factor for autistic-like behavior in rats [43]. The development of VAD during pregnancy can lead to impaired learning and memory in offspring [44]. These findings suggest that VAD may affect brain development and especially cause alterations in the hippocampus, which can lead to ASD-associated symptoms and ultimately the development of ASD (Figure 1).

### 4.2. VAD in the Digestive System of ASD

The GI tract, known as the human second brain, is considered to have an important role in ASD, as the microbiota-gut-brain axis is thought to be involved in the pathogenesis of ASD [45]. In addition to its core symptoms, ASD often presents with GI symptoms, such as diarrhea, constipation, abdominal pain, nausea or vomiting, and flatulence [46, 47]. Constipation is the most common GI symptom in ASD children [47, 48]. Besides GI symptoms, several studies have shown that intestinal bacteria are altered in ASD children [49–52]. Compared with TD children, *Clostridium* [15], *Sutterella* flora, *Lactobacillus*, and *Desulfovibrio* were found to be increased in ASD children, while Bacteroidetes and Firmicutes were decreased, and these changes were closely related to the severity of ASD [51, 53]. Antibiotics have been found to improve autistic-like behavior in ASD animal models [54, 55]; social disorders can be observed in germ-free mice [56], while probiotic intervention in germ-free mice can promote social behavior [57]; probiotic treatment with *Bacteroides fragilis* can improve autistic-like behavior in animal models of ASD [57, 58]. In conclusion, GI symptoms and intestinal microflora are closely related to autistic-like behavior. However, it is not clear whether GI symptoms or intestinal microflora disorder can lead to ASD. More scientists in the field believe that GI symptoms and intestinal microflora have an effect on each other. Also, several studies have shown that VA is related to GI diseases [59, 60]. Indeed, RA is an important factor in promoting intestinal immunity [61] and maintaining mucosal epithelial integrity [62]. It has also been shown that RA can promote changes in intestinal bacterial composition in ASD children and has some benefits in increasing the autism biomarkers CD38 and acid-related orphan receptor (RORA) [60]. The above evidence suggests that VA, GI symptoms, and the changes in the intestinal bacterial community are closely related to ASD. Animal studies have shown that VA can control lymphoid tissue [63], relieve diarrhea, and improve intestinal injury [64], while VAD can disrupt the intestinal physical barrier function [65]. Some studies have shown that a VA-deficient diet leads to changes in intestinal bacteria in rats [39], and RA can reduce the proportion of Firmicutes and Bacteroidetes in animals [40]. Therefore, VA has the potential to be associated with ASD through GI function and regulation of intestinal bacteria, and this notion is supported by additional current relevant findings. The results of a population study suggest that VA levels are strongly associated with the development of GI symptoms, especially constipation, in ASD children [66]. VAD aggravates core symptoms of ASD children, and ASD children with GI symptoms also have more severe core symptoms than those without GI comorbidity. The coexistence of VAD with GI significantly exacerbates core symptoms in ASD children. Moreover, a study in a valproic acid- (VPA-) induced rat model of autism in pregnant rats with VAD conducted to investigate the relationship between VAD, ASD, and GI comorbidities [67] showed that VPA induced an increase in GI transit time and intestinal nervous system damage in ASD rats. Compared with ASD rats with normal VA, VA-deficient pregnant ASD rats showed more autism-like behaviors (especially social dysfunction), and VAD aggravated impairment of GI motility and the enteric nervous system (ENS). Additionally, VA supplementation was found to improve autism-like behavior and ENS dysplasia in VA-deficient pregnant ASD rats. In conclusion, VAD may be involved in the development and progression of ASD by affecting GI function or regulating intestinal flora (Figure 1).

### 4.3. VAD in the Immune System of ASD

Multiple studies have shown that VA plays an important role in the immune system. The level of IL-6 in the brain of children with ASD has been found to be significantly increased. The increased expression of IL-6 can lead to changes in the adhesion and migration of nerve cells and cause an imbalance of excitatory and inhibitory circuits [68]. In addition, the concentrations of IL-6, TGF-*β*, IL-17, and IL-10 were significantly higher in gut-associated lymph node tissue and draining lymph nodes of VA-deficient mice, suggesting the presence of inflammatory lesions in the intestine, which were significantly improved after VA supplementation. This may be due to VAD-induced oxidative damage, leading to the loss of rapid mitochondrial membrane potential, while supplementation with physiological doses of VA may reverse this effect [69]. This finding suggests that VAD may affect the development or symptoms of ASD by regulating the immune system through inflammatory cytokines like IL-6.

Oxytocin (OXT) is a neuropeptide, produced by the hypothalamus and secreted by the posterior pituitary gland, which can be used as a molecular marker for assessing social function [70]. Studies have shown that OXT nasal spray increases brain activity and clearly improves social functioning in ASD children [70]. OXT can promote maternal behavior, social memory, and social connections in mammals [71]. In one study, plasma OXT levels in eight 18-year-old ASD individuals were significantly lower than those in TD individuals [72]. Another study showed that the OXT receptor gene (OXTR) is associated with susceptibility to ASD [73]. In addition, intranasal or subcutaneous administration of OXT was reported to improve social cognition, coordination, reciprocity, and empathy and reduce repetitive behaviors in ASD patients and mice [74–77]. The link between OXT and ASD has received considerable attention, and as such, OXT has been extensively studied [74, 75].

CD38 is a transmembrane glycoprotein that can increase calcium excitability and promote OXT expression in hypothalamic neurons *via* ADP-nucleoside cyclase activity [76–79]. Many studies have shown that the CD38-OXT pathway is significantly associated with ASD [79–81]. CD38 expression was found to be lower in lymphoblastoid cell (LBC) lines from ASD patients than in parental lines [82]. ATRA, a major inducer of CD38 expression [83], was shown to upregulate CD38 expression in ASD LBC lines [84]. In addition, CD38 levels were found to be low in lipid cells extracted from ASD patients but increased after the addition of RA [83]. The above results suggest that VAD may be linked to ASD by affecting the CD38-OXT axis. A VAD or VA-supplemented (VAS) animal model was established in pregnant rats and used to study whether VAD is a risk factor for autistic-like behavior [85]. The study revealed that VAD in pregnant rats exacerbated autistic-like behavior and reduced the expression of RAR-*β* and CD38 in the hypothalamus and serum OXT levels in rat offspring. These indicators were improved after VA supplementation. Moreover, it was found to enhance ATRA, the binding of RAR-*β* to the CD38 promoter. This suggests that VAD during pregnancy may inhibit the expression of CD38 in the hypothalamus of rat offspring by affecting the binding of RAR-*β* to CD38, thereby inhibiting the release of OXT and ultimately exacerbating autistic-like behavior (Figure 1). Early VAS was found to improve such conditions. The above studies suggest that VA may be involved in the etiology or symptomatology of ASD by affecting the immune system of ASD patients, for example, through IL-16 and CD38 (Figure 1).

### 4.4. VAD in the Serotonin System of ASD

Serotonin, also known as 5-HT, plays an important role in regulating the physiological activities of the central and peripheral nervous systems. Tryptophan hydroxylase (TpH) is the first rate-limiting enzyme in 5-HT synthesis and can control the synthesis and metabolism of 5-HT, including the two subtypes TpH1 and TpH2, which are involved in the synthesis of 5-HT in the peripheral and central nervous systems, respectively [86, 87]. Some studies have shown that children with ASD have increased 5-HT, leading some researchers to believe that ASD is related to the 5-HT neuronal system. In TD children, the 5-HT synthesis capacity is high before the age of 5 years and gradually decreases to adult levels after the age of 5, whereas in children with ASD, from the age of 2 to 15 years, 5-HT synthesis capacity still gradually increases and can reach 1.5 times that in normal adults. Several studies have found significantly higher plasma and serum 5-HT levels in ASD children compared to TD children [88, 89]. One study showed that about one-third of ASD patients have hyperserotonemia, and 5-HT levels are associated with self-mutilation behavior [90]. In addition, researchers have also found a positive correlation between the peripheral blood 5-HT levels and the severity of ASD [91]. In animal experiments, motor and cognitive impairments resulting from developmental hyperserotonemia may be related to the neuropathological and functional/behavioral alterations observed in ASD [92]; thus, 5-HT levels are closely related to ASD. The results of another study showed that 5-HT levels significantly decreased in ASD children after VA supplementation, which improved symptoms in ASD children [93]. Considering that the expression level of TpH1 mRNA in peripheral blood leukocytes was significantly decreased after VA supplementation, while the expression level of TpH1 mRNA in peripheral blood leukocytes of ASD children was significantly higher compared with that of the control group, and considering the possible presence of RAR-*γ* binding sites for TpH1, VA supplementation may regulate the synthesis of 5-HT through RAR-regulated TpH1 expression. The above results indicate that VAD may affect ASD by regulating 5-HT levels, and 5-HT may serve as a biomarker for ASD (Figure 1).

## 5. Role of VA in ASD Treatment

Currently, information on the effectiveness of VA in treating ASD is preliminary and limited. Numerous studies have shown a potential protective effect of RA on the neurodevelopment of ASD. First, RA has been shown to protect neurons from oxidative stress and inflammation [94, 95], and the increases in oxidative stress and inflammatory markers may contribute to the development of ASD [96, 97]. Second, somatostatin, which is associated with cognitive function, as well as another potential mechanism for altered neuronal processing that may be related to somatostatin, has been shown to be severely reduced in the setting of VAD. Third, the brain region with high levels of RA signaling is the striatum. RXR (retinoid X receptor) ligands may control dopaminergic neuron development and induce neuronal regeneration [38]. Fourth, studies have shown that dysfunction of the GABAergic signaling pathway is involved in the pathogenesis of ASD [98], and RA can affect the migration of neurons from ganglion processes to the cerebral cortex and provide GABA to interfere with neuron firing [99]. Finally, CD38 and RORA are critical in social behavior in ASD [99–101]. CD38 and RORA mRNA levels have been found to be significantly increased in the serum of ASD patients after VA intervention [59]. Earlier studies have also found that RA can upregulate the transcription level of CD38 [57, 102]. In addition, RA may also regulate RORA, RORB, and RORR through its RA receptors, which suggests a potential role for VA in the treatment of ASD [103].

In 2000, Megson described the potential application of VA in the treatment of ASD as well as the need for clinical trials [104]. Studies have shown that the Bacteroides/Bacteroides and Bacteroidetes/Firmicutes ratios are reduced in ASD, and Bacteroidetes intervention can improve social behavior in ASD patients [50, 105, 106]. A study in Hainan Province showed that social interaction was improved in ASD children after VA supplementation [19]. A 6-month follow-up study of 64 ASD children aged 1–8 years found that after VA intervention, Bacteroidetes increased and ASD-associated CD38 and RORA mRNA levels increased [59]. A study of 33 ASD children (mean age: 5.14 ± 1.33 years) and 32 TD controls conducted for 6 months to determine whether VA supplementation improves autistic-like symptoms [93] found that serum retinol levels were significantly lower in ASD patients than in controls. Moreover, serum retinol levels were significantly increased in ASD children after VA supplementation. These results showed that ASD children synthesized more 5-HT than TD children, and VA supplementation could reduce the synthesis of 5-HT in ASD children, thereby improving their symptoms. The above population studies showed that after VA intervention, autistic-like symptoms were improved in ASD children compared to TD children. In addition, animal experiments have shown that VA supplementation improves autistic-like behavior and ENS dysplasia in ASD rats [20]. Recently, a study designed to investigate a better VAS program in 138 3–8-year-old children with ASD [107] found that VAS improved serum VA and alleviated the social impairment of ASD children, likely due to the improvement of the expression of the RAR-*β*-CD38-OXT axis in ASD children after VAS intervention.

In summary, results from numerous studies show that VA supplementation may improve, to a certain extent, the symptoms of ASD patients by influencing neurodevelopment, intestinal bacteria, immune markers, and other processes. Although there is still a lack of a unified and effective treatment regimen for the treatment of ASD, micronutrient supplementation, including VA, has the advantages of fewer side effects on the human body, high patient acceptance, convenient and inexpensive sources, etc. Therefore, studies on the efficacy and understanding of the specific mechanism of VA supplementation in ASD patients and even clinical application of expanded research deserve further attention and investment.

## 6. Conclusions

The rapid increase in the prevalence of ASD is increasingly harmful to families and society. VAD is one of the most prevalent micronutrient deficiencies in the world and is most common in developing countries, especially in children and pregnant women. In recent years, numerous studies have confirmed that VAD is one of the risk factors for ASD and suggested that VAD may be involved in the pathogenesis of ASD, and VA supplementation may represent a potential treatment for ASD.

Several studies have found that VA and VD have similar effects on ASD. In the nervous system, deficiency of VA and VD can affect nerve growth factors, synaptic function, brain development, and maturation processes and ultimately lead to impairment of related memory formation and behavior. In the digestive system, changes in the vitamin A or D status affect the gut microbiota. The major microbial phyla in humans and rodents are Firmicutes and Bacteroidetes, which play an important role in ASD [108]. Furthermore, both VA and VD are fat-soluble vitamins with some shared chemical properties. Therefore, it is reasonable to suggest that there is a partial overlap in the roles of VA and VD in ASD. In addition, we can compare and learn from the studies of VA and VD in children with ASD and even investigate the effect of VA and VD combination therapy on ASD children to further understand the effects of micronutrients, especially those of VA and VD, on ASD children. However, additional research is necessary to address these questions about the etiology and symptomatology of ASD, as well as the therapeutic role and mechanism of VA in ASD. The data presented in this review can provide important guidance for future related research on VA and ASD.
